# Hybrid Dissolved-Oxygen and Temperature Sensing: A Nanophotonic Probe for Real-Time Monitoring of Chlorella Algae

**DOI:** 10.3390/s21196553

**Published:** 2021-09-30

**Authors:** Niloofar Fallahi Chegeni, Parto Ijadi Maghsoodi, Mahsa Habibi, Hossein Zare-Behtash, Mohammad Hossein Majles Ara, Esmaeil Heydari

**Affiliations:** 1 Faculty of Physics, Kharazmi University, Tehran 15719-14911, Iran; std_Niloofarfalahi@khu.ac.ir (N.F.C.); parto.ijadi.maghsoodi@vub.be (P.I.M.); std_mahsa.habibi@khu.ac.ir (M.H.); majlesara@khu.ac.ir (M.H.M.A.); 2 James Watt School of Engineering, University of Glasgow, Glasgow G12 8QQ, UK; Hossein.Zare-Behtash@glasgow.ac.uk; 3 Research Affiliate, James Watt School of Engineering, University of Glasgow, Glasgow G12 8QQ, UK

**Keywords:** dissolved oxygen, temperature sensing, lanthanide-doped upconversion nanoparticles, platinum porphyrin

## Abstract

Dissolved-oxygen concentration and temperature are amongst the crucial parameters required for the precise monitoring of biological and biomedical systems. A novel hybrid nanocomposite probe for real-time and contactless measurement of both dissolved-oxygen concentration and temperature, based on a combination of downconverting phosphorescent molecules of platinum octaethylporphyrin and lanthanide-doped upconverting nanoparticles immobilized in a host of polystyrene, is here introduced. Chlorella algae are employed here as a model to demonstrate the hybrid nanophotonic sensor’s capability to monitor the aforementioned two parameters during the photosynthesis process, since these are among the parameters impacting their production efficiency. These algae have attracted tremendous interest due to their potential to be used for diverse applications such as biofuel production; however, feasibility studies on their economic production are still underway.

## 1. Introduction

Oxygen concentration and temperature influence a great majority of biological processes. Oxygen is a vital analyte required by the majority of living creatures on Earth [1]. It plays a crucial role in diverse fields of research and various industries such as life sciences, biotechnology, the food industry, waste-water management, oil and petrochemicals, space exploration, and oceanography [2]. Moreover, oxygen is a key element in studying pathological and physiological processes; thus, measuring the dissolved oxygen (DO) both in and outside of living bodies is essential to understand the processes involved [3,4]. Various sensing mechanisms have been introduced for DO detection, in which the Clark electrode is the most common commercial sensor to be used [5]. However, Clark sensors suffer from several drawbacks such as slow response times, large size, the requirement for regular calibration and replacement of electrolyte and permeable cap, and consumption of oxygen during measurements, which is detrimental in low-level DO detection. There is a growing interest in the application of dynamic photoluminescence (PL) quenching sensors based on molecular oxygen-sensitive probes (OSP) for DO detection [6,7]. This results from properties such as high sensitivity and selectivity, non-invasiveness, contactless, reversibility, capability to become very small, and real-time detection, such that they have met the stringent conditions necessary for NASA’s space cell culture analyses [8]. Therefore, the PL-based OSPs have the potential to take over the traditional market of Clark electrodes. Popular luminescent OSPs include organic compounds of Ruthenium (II), Iridium (III), Platinum (II), and Palladium (II). PL in these organometallic compounds is the result of charge transfer from the metal-ligand band to the ground state where they are excited. In these OSPs, PL intensity and lifetime quench reversibly in the presence of oxygen molecules, enabling the measurement of oxygen partial pressure [9,10,11]. These OSPs are usually doped in oxygen-permeable polymer hosts, in different forms and shapes, to become immobilized and avoid contamination by leaching into the understudying environment. Earlier investigations showed that the oxygen sensor’s performance, marked by the Stern–Volmer plot (SVP) sensitivity and linearity, depends on selecting the hosting matrix. This matrix needs to show a high level of permeability to oxygen and be impermeable to other substances. In addition, resistance to photo-oxidation, mechanical and chemical stability, and flexibility are desirable. To this end, fluorine-based matrices have recently been used [12,13]. The selection of photoluminophores with long lifetimes, whilst increasing the sensitivity, reduces the vulnerability to polymer matrices [14]. Platinum octaethylporphyrin (PtOEP) is among the high-performance OSP suitable for DO sensing due to its significant Stokes shift. It also demonstrates a longer PL lifetime and quenching efficiency than the other widespread OSPs such as Ruthenium complexes. In addition, it has also been adopted for DO measurement in a micro-scale environment [15]. It has been reported that encasing the OSPs in silica nano-shells enhances their sensitivity by increasing the surface area per unit mass [16].

The value of temperature measurements is already well established [17,18]. There have been two approaches developed for temperature sensing, namely, contact and non-contact methods. The contact approach is limited by electrical wiring, whereas the non-contact method, based on PL measurement, has attracted greater attention in biological/biomedical applications. In the last decade, Er^3+^-based upconversion nanoparticles (UCNPs) were introduced as a reliable ratiometric temperature nanosensor because of their significant PL change in the physiological temperature range [19]. A specific spectral band of NaYF_4_:Yb^3+^, Er^3+^ is temperature sensitive so that its work function changes linearly with increasing temperature [20]. UC nano-emitters convert two or more low-energy photons in the near-infrared (NIR) spectrum into a high-energy photon in the visible spectrum through a nonlinear phenomenon [21]. Typically, they are excited using a 980 nm NIR laser, and their PL emission lies in the visible spectrum [22]. Furthermore, they exhibit unique features such as a significant Stokes shift, long lifetime, and spectrally narrow emissions.

Photosynthesis is a fundamental biological process upon which life on earth, either directly or indirectly, depends. Microalgae hold an essential role in the first level of the food chain in lakes and rivers. They are the most common oxygenic organisms in the aquatic ecosystem, which have evolved for the past 3 billion years to exploit the Sun’s abundant energy by relying on their light-harvesting complex proteins. This process is the driving force for consuming CO_2_ and converting it to products such as lipid-rich biomass, protein, starch, and O_2_. These single-celled green microalgae have recently attracted attention for applications such as generating renewable energy, sequestering CO_2_, wastewater treatment, health products, fertilizers, and food production [23]. For the last century, the growing concern of global warming and the energy crisis has led to significant investigations into the identification and development of sustainable energy sources. Microalgae are a promising candidate for future biofuels, since they are efficient in converting CO_2_ gas into carbon-rich lipids, which are a precursor to biodiesel fuel [24,25,26,27]. In addition, microalgae-based processes for biological nutrient removal (BNR) in wastewater have been introduced as an economically and environmentally alternative method to the conventional processes, because they can utilize nutrients of wastewater and use CO_2_ from greenhouse gases for photosynthesis [28].

In the current study, a novel hybrid nanophotonic probe based on PtOEP organometallic complex and NaYF_4_:Yb^3+^, Er^3+^ upconversion nano-emitters doped in a polystyrene (PS) matrix are implemented for real-time and contactless monitoring of DO concentration and temperature in a Chlorella algae culture medium.

## 2. Materials and Methods

Fabrication of temperature-oxygen sensing probe (TOSP): PtOEP was purchased from Lumtech in Taiwan. Toluene and PS were purchased from Sigma Aldrich. A solution of TOSP was prepared by dissolving 2 mg of PtOEP powder in 2 mL of Toluene. Afterward, 240 mg of PS matrix was added to the solution in order to prepare a film. In the next step, 300 μL of 38 mg/mL NaYF_4_:Yb^3+^, Er^3+^ TSP colloidal solution was added to the solution. The final solution was treated on a magnetic stirrer for 1 h to make a homogeneous solution. Thick films were produced by drop-casting of 300 μL of the final solution onto a 1.5 cm × 1.5 cm clean glass slide and baking it for 1 h in a 100 °C oven.

Lifetime Measurement: Nd:YAG laser with a wavelength of 532 nm and pulse width of 12 nm from Parto Pardazesh Mavad Tehran was used for the excitation. Time-resolved PL measurement was performed using Thorlabs PDA10A2 photodetector, Thorlabs NF533-17 notch filter, and Tektronix TDS3052B oscilloscope. DO measurement was carried out using the HANNA edge HI2040 Clark electric oxygen meter. Temperature Measurement: 980 nm laser from CNI was used for excitation of TOSP. PL measurement was used employing a CCS100 Thorlabs spectrometer. Temperature reference measurement was carried out using a DS18B20 waterproof temperature sensor from Dallas semiconductor in combination with Arduino UNO.

## 3. Results

**Figure 1:**
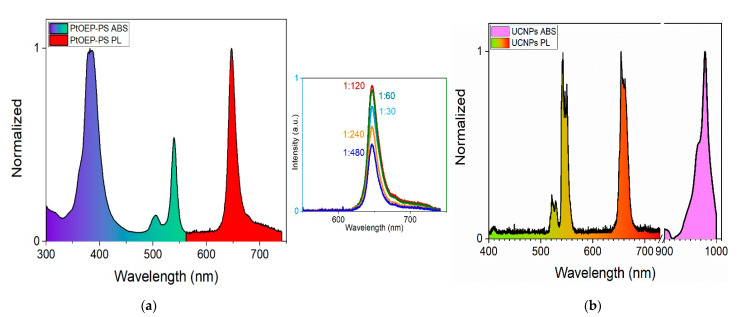
(**a**) Absorption (ABS) and emission spectra (λ_ext_ = 532 nm) of PtOEP doped in PS matrix. Inset is the PL intensity for different PtOEP to Ps ratios; (**b**) PL of PtOEP-PS/NaYF_4_:Yb^3+^, Er^3+^ nanocomposite film (λ_ext_ = 980 nm) and absorption of NaYF_4_: Yb^3+^, Er^3+^ nano-emitters.

PtOEP was selected as the OSP due to its excellent oxygen sensitivity, sufficient emission intensity, high solubility, long lifetime, and relatively significant Stokes shift. NaYF_4_:Yb^3+^, Er^3+^ UC nano-emitters were synthesized as described by Kaboli et al. [29] and employed as the temperature-sensitive probes (TSP) due to their linear temperature response, significant Stokes shift, spectrally narrow emission in the visible spectrum, NIR excitation, and their lack of oxygen sensitivity [30]. Different weight proportions of PtOEP to PS were prepared to determine the optically efficient concentration for DO measurement. Figure 1a depicts the absorption and PL emission spectra of a PtOEP organometallic complex doped in the PS matrix. PL results, inset figure, demonstrates that the weight ratio of 1:120 is the optimum ratio. The absorption spectrum is indicated with ultraviolet-green color and PL emission with the color red. The peak at 385 nm is associated with the Soret band, and the two other peaks at 505 nm and 535 nm are associated with the Q-band. The PL spectrum exhibits a maximum at 648 nm when excited with a 532 nm Nd:YAG laser. A Stokes shift greater than 100 nm was observed for the PtOEP-PS film. NaYF_4_:Yb^3+^, Er^3+^ nano-emitters were doped in PtOEP-PS solution to prepare the TOSP. Figure 1b illustrates the absorption spectrum of the NaYF_4_:Yb^3+^, Er^3+^ UC nano-emitters, indicated with a light pink color, and PL spectrum of the PtOEP-PS/NaYF_4_:Yb^3+^, Er^3+^ nanocomposite film, indicated with a green-red color when it is excited with a 980 nm laser beam. There are three main peaks at 521 nm, 541 nm, and 654 nm associated with the PL emission of the nanocomposite. The maximum absorption of the UCNPs is at 978 nm, which is used for optical excitation using a 980 nm laser beam.

**Figure 2:**
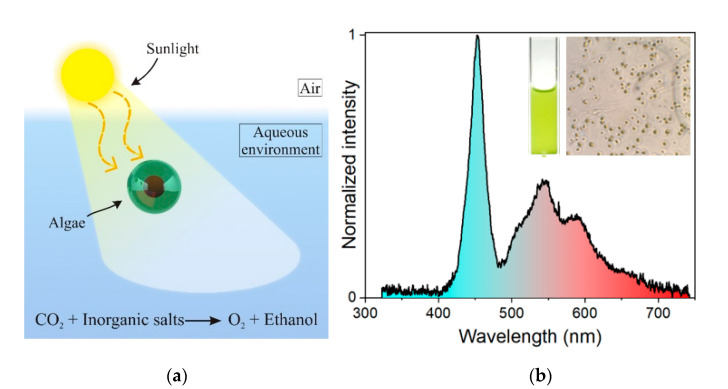
(**a**) Schematic illustration of the photosynthesis process by Chlorella microalgae; (**b**) emission spectrum of LED array used for photosynthesis of the Chlorella microalgae. Inset are the images of microalgae in a cuvette and under the optical microscope.

TOSP was implemented for Chlorella microalgae monitoring to demonstrate its capability for real-time and contactless monitoring of the oxygen consumption/generation in the culture medium during a long-term photosynthesis process. The schematic of photosynthesis by microalgae is illustrated in Figure 2a. The emission spectrum of the employed LED array is shown in the inset of Figure 2b. The illuminance of the LED array was 24,000 Lux measured by Dr. Meter LX1330b. Images of the microalgae in a cuvette and under the optical microscope are shown in the inset.

### 3.1. Real-Time Monitoring of DO Concentration

**Figure 3:**
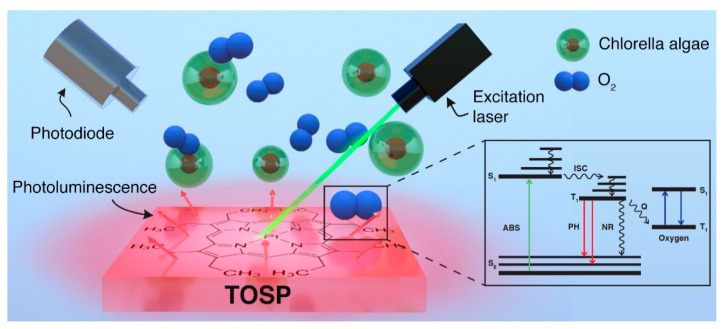
Schematic demonstrations of oxygen detection mechanism.

Figure 3 schematically illustrates the mechanism involved in the DO sensing. Initially, the molecular PtOEP in TOSP was optically excited from its ground state to higher energy states by 532 nm laser pulses. The wavelength of the excitation laser lies in the absorption spectrum of the PtOEP. Subsequently, excited PtOEP molecules release part of this energy radiatively in the form of PL, mainly phosphorescence, and lose some part non-radiatively due to collisions with oxygen molecules. Thus, the PL lifetime and intensity are a function of the DO concentration, and increasing this concentration leads to more collisions between the excited PtOEP molecules in the TOSP and oxygen molecules resulting in a higher level of quenching of PL lifetime and intensity. The lifetime-based DO detection is more favorable over intensity-based DO detection, since it is less vulnerable to photobleaching and environmental interference and is more accurate.

**Figure 4:**
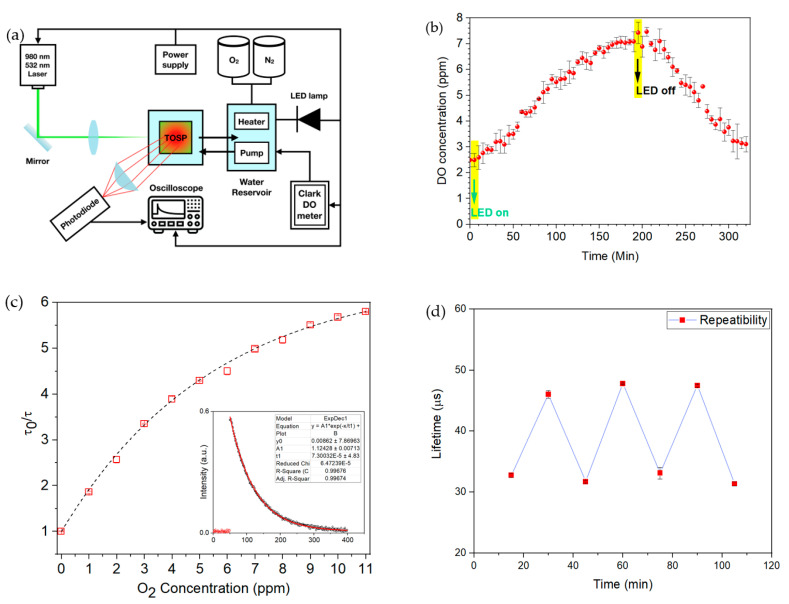
(**a**) Optical setup for hybrid DO and temperature measurements; (**b**) DO concentration versus time for the microalgae culture medium when the LED was turned on and off; (**c**) lifetime ratio versus oxygen concentration and inset is a typical PL decay curve fitted with an exponential function to achieve the PL lifetime; (**d**) repeatability of the TOSP when the oxygen concentration is varied between 0.5 and 1.5 ppm.

First, DO measurement for a water sample was performed, using a reference DO sensor to calibrate the hybrid TOSP. Figure 4a demonstrates the experimental setup that was used for the calibration of the TOSP. A water chamber was made from transparent plexiglass, then a 1 mm thick film of the TOSP was fixed with screws in this chamber. The DO concentration in the chamber was controlled by a combination of N_2_ and O_2_ gases. Their ratio was tuned using a Lematec digital regulator. The TOSP was optically excited using 532 nm laser pulses with a pulse width of 12 ns and spot diameter of less than 1 mm. A 5 cm convex lens collected the PL emission after passing through a Thorlabs NF533-17 notch filter focused on a Thorlabs PDA10A2 photodetector connected to a Tektronix TDS3052B oscilloscope. A HANNA edge HI2040 Clark electric oxygen meter was used as a reference DO meter in the experiments. A PL lifetime ratio at zero DO concentration (used as reference state) to the PL lifetime at other DO concentrations 𝜏_0_/𝜏 was measured when the TOSP was placed in the aqueous chamber, where the water was pumped by an R385 diaphragm pump from a 2 L reservoir with the flow rate of 1.2 $\frac{L}{min}$. At first, the DO concentration was reduced to zero by injecting the N_2_ gas into the aqueous chamber. Afterward, the time-resolved PL measurement was carried out when the DO concentration gradually increased from zero in order to calibrate the sensor. The ratio of 𝜏_0_/𝜏_100_, which is an indication of the sensitivity, is 9.7. In the next step, the TOSP was used for monitoring the DO parameter in a microalgae culture medium. The TOSP was excited from the rear of the chamber; therefore, the laser light did not go through the algae medium to interact with the TOSP. In this experiment, the culture medium was kept in the dark room overnight, then the LED light was turned on, and the lifetime of the TOSP was measured every 5 min for 5.5 h at a constant temperature with a flow rate of 1.2 $\frac{L}{min}$. The real-time monitoring of oxygen generation initiated by an LED light source is depicted in Figure 4b, in which initially, the PL lifetime was decreased, and correspondingly, the oxygen concentration increased while the light was on for 3.25 h. The lifetime quenching is attributed to oxygen generation by the photosynthesis process. Photosynthesis reduces the lifetime of the TOSP by increasing the non-radiative energy transfer from the excited PtOEP molecule to the produced oxygen molecules. Afterward, the light was turned off immediately, and the lifetime measurement was carried out for another 2.25 h. Figure 4b illustrates that the lifetime begins to increase, since the oxygen production induced by photosynthesis is stopped. Therefore, the non-radiative energy transfer to the oxygen molecules was reduced by depleting the oxygen production, which subsequently appears as an increase in the lifetime of the TOSP.

Figure 4c demonstrates the 𝜏_0_/𝜏 ratio based on the DO concentration. Ideally, the relationship is linear; however, in actual scenarios, it may deviate from the linear behavior when TOSP is doped in a polymer matrix [31]. Figure 4c inset shows a typical PL decay fitted with a one-term exponential function using the Levenberg–Marquardt algorithm to achieve the corresponding lifetime when the TOSP is excited with the laser pulses. The repeatability of the TOSP was evaluated as 97% by repetitive measurement of oxygen concentration. For instance, Figure 4d shows the repeatability test for oxygen change in the range of 0.5 to 1.5 ppm.

### 3.2. Real-Time Monitoring of Temperature

**Figure 5:**
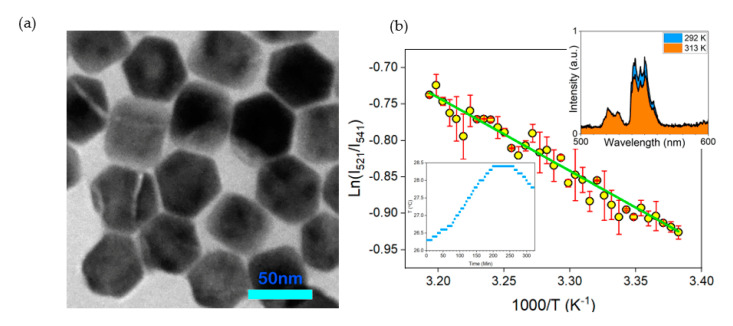
(**a**) TEM image of NaYF_4_:Yb^3+^, Er^3+^ lanthanide–doped UCNPs; (**b**) linear fitting of natural logarithm of intensity ratio at 521 nm and 542 nm as a function of temperature, inset top right: PL spectrum of the upconverting nanocomposite at two different temperatures; inset bottom left: temperature measurement based on UCNPs.

NaYF_4_: Yb^3+^, Er^3+^ UCNPs doped in PtOEP-PS, was employed as a TOSP for real-time temperature measurements. In these nano-emitters, Yb^3+^ ions are used as sensitizers, and Er^3+^ ions are activators. The Yb^3+^ ions provide a single excited state for subsequent accumulation of NIR photons and efficient energy transfer to the Er^3+^ ions to emit of a single photon in the visible spectrum. The TOSP was pumped with a 300 mW laser with a wavelength of 980 nm. The PL emission was focused on a 600 μm Avantes optical fiber by a 5 cm convex lens and transferred to a CCS100 Thorlabs optical spectrometer for spectral analysis. A temperature-controlled Peltier was used to increase the temperature. A DS18B20 waterproof temperature sensor from a Dallas semiconductor with a resolution of 0.06 °C combined with Arduino UNO was employed as the reference temperature sensor. Figure 5a shows the TEM image of the UCNPs captured using a Philips EM208S 100 KV microscope. The average diameter of the hexagonal NPs is in the order of 50 nm. Ratiometric temperature measurement was performed by selecting coupled wavelengths of 521 nm and 541 nm. The intensity ratio is typically plotted against the inverted temperature. Figure 5b depicts the I_521_/I_541_ graph based on the corresponding inverted temperatures multiplied by 1000. The behavior of the TOSP is fitted with a linear function. Therefore, the maximum temperature-dependent sensitivity is 0.0049 at 313 K. It was observed that by increasing the temperature of the chamber, the main emission band at the shorter wavelength of 521 nm was slightly varied or remained constant while the second main emission band at the higher wavelength of 541 nm decreased, as it is presented in the inset of Figure 5b on the right corner. The PL spectrum at the lower temperature (292 K) is colored in blue and, at the higher temperature (313 K), is colored in orange. Thus, the UC efficiency is temperature-dependent, and it can be used as a TOSP for nanophotonic-based temperature sensing. Finally, the TOSP was employed for monitoring the temperature variation in microalgae culture medium The temperature increases from 26.4 °C to 28.4 °C, as illustrated in Figure 5b inset in the left corner.

## 4. Conclusions

In this paper, for the first time, a hybrid nanophotonic probe consisting of an organometallic platinum complex and lanthanide-doped upconversion nanoparticles was employed for the remote, contactless, and real-time detection of oxygen concentration and temperature in an aqueous medium. The platinum complex was used for optical dissolved-oxygen sensing and NaYF_4_:Yb^3+^,Er^3+^ nano-emitters for temperature sensing. In recent years, chlorella microalgae have found considerable attention due to their potential to become a source of future fuel, food supplements, water waste treatment, and CO_2_ management. The sensor was employed for real-time and remote monitoring of photosynthesis-based oxygen generation and natural depletion, as well as temperature changes in a chlorella microalgae medium. The oxygen level was measured by fitting the photoluminescence decay curve with an exponential function. The temperature was determined from the ratiometric photoluminescence intensity. Results support that this hybrid sensor is suitable for monitoring oxygen and temperature changes in an aqueous medium instead of Clark electrodes with the added benefits that the probe size is much smaller, in the order of the laser spot diameter, it does not require repetitive calibration, and the measurement is contactless and continuous.

## Data Availability

Data are contained within the article.
